# Ergonomists’ experiences of executing occupational health surveillance for workers exposed to hand-intensive work: a qualitative exploration

**DOI:** 10.1186/s12913-022-08601-2

**Published:** 2022-10-01

**Authors:** Kristina Eliasson, Anncristine Fjellman-Wiklund, Gunilla Dahlgren, Therese Hellman, Magnus Svartengren, Teresia Nyman, Charlotte Lewis

**Affiliations:** 1 Department of Medical Sciences, Occupational and Environmental Medicine, Uppsala University, Uppsala, Sweden; 2 Department of Occupational and Environmental Medicine, Uppsala University Hospital, 751 85 Uppsala, Sweden; 3 Department of Community Health and Rehabilitation, Physiotherapy, Umeå University, Umeå, Sweden; 4 Department of Public Health and Clinical Medicine, Section of Sustainable Health, Umeå University, Umeå, Sweden

**Keywords:** Ergonomics, Legislation, Medical health checks, Qualitative research, Risk assessment, Work environment, Work-related upper limb disorders, Sweden

## Abstract

**Background:**

In order to reduce work-related upper limb disorders, the Swedish Work Environment Authority introduced an occupational health surveillance targeting hand-intensive work. A process model, aimed at supporting the employers as well as the occupational health service provider (i.e., ergonomist) in the work process with the occupational health surveillance, was developed. The objective of this qualitative study was to explore ergonomists’ experiences of the execution of occupational health surveillance for hand-intensive work when following the novel process model as well as factors influencing the execution.

**Methods:**

Semi-structured individual interviews were conducted with ten ergonomists on one occasion regarding their experience of following the work process. Qualitative content analysis with an inductive approach was used for analyzing the data.

**Results:**

The ergonomists’ experiences were summarized in one theme “A joint roadmap supporting a participatory process*”* and two categories “Clear structure provided by the components” and “The process influenced by collaboration and context”. The ergonomists valued being guided by the systematics of the model, which provided structure and clarity in their work. Factors affecting the execution were related to communication deficiencies and uncertainties regarding expectations between different roles and functions (e.g., ergonomists and contact person, lack of information to workers). Additional factors, for instance, companies’ routines and the ergonomist’s intra-organizational support, such as access to IT-resources, could also affect the process.

**Conclusions:**

The findings reveal that this process model facilitates the ergonomists’ work and cooperation with a client company. However, the process model needs to be developed and accompanied by a guideline with information related to the process, including e.g., description of a start-up meeting and of the roles/functions of the involved parties.

**Supplementary Information:**

The online version contains supplementary material available at 10.1186/s12913-022-08601-2.

## Introduction

Work-related upper limb disorders (WRULD) are a well-known problem worldwide, causing work disability, productivity loss, and societal costs [1–3]. In Sweden, in 2020, 58% of those who claimed that they suffered from ill health during the last twelve months due to conditions at work, other than an accident, reported symptoms in the neck and/or upper extremities [4]. These disorders are associated with hand-intensive work, which involves forceful exertion, high repetition, long duration, awkward or static postures, and often combinations of these characteristics [5–11]. Hand-intensive work is commonly in many sectors, for example; different assembly work, food industry (processing and packing), construction and interior work (painters, carpenters etc.). By identifying and reducing hazardous exposures, WRULDs can be prevented among workers exposed to hand-intensive work.

A legislative incentive can prevent work-related injuries and disorders [12, 13], and both general and specific legislation are effective in reducing fatalities and injuries and improving the work environment [14]. In 2019, the Swedish Work Environment Authority revised the legislation concerning occupational health surveillance and introduced a specific occupational health surveillance, targeting hand-intensive work [15]. The goals of the occupational health surveillance are: (1) restrict exposure for sensitive individuals (removing the individual from the work task), (2) early detection of work-related ill-health, and (3) provide information for measures targeting the work environment in the workplace ([15, 16], chapter 6). According to the revised Swedish legislation, a person who executes the occupational health surveillance targeting hand-intensive work should have one of the following competences: Physician (MD), registered physiotherapist (RPT), licensed naprapath or licensed chiropractor. Moreover, the person needs to have:

1) sufficient knowledge of work environment management, 2) sufficient knowledge of the employee's exposure and working conditions, 3) clinical competence for examination of the musculoskeletal system, and 4) competence to assess whether the hand-intensive work can cause disorders in neck, shoulder, arm or hand [15].

**Fig. 1:**
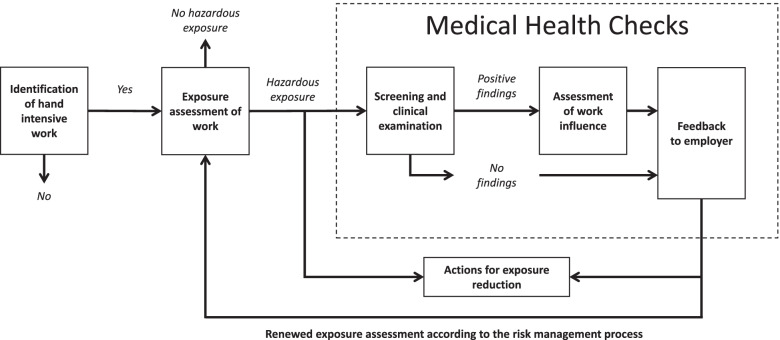
The hand intensive work-model for periodical occupational health surveillance for workers exposed to hand intensive work

As a result of the new legislation, a novel process model was developed for occupational health surveillance of workers exposed to hand-intensive work (the HIW-model). The development of the model as well as the description of its components have been presented by Eliasson et al. [17]. In order to be recognizable and enable integration in a company’s occupational health and safety management (OHSM) system, the HIW-model is structured according to the Plan-Do-Act-Check process and includes the core components of a risk management process: identification, assessment, control, and monitoring of workplace hazards [18, 19]. The model is illustrated below (Fig. 1) and aims to guide the employer (as having the legal responsibility for the work environment) and the Occupational Health Service (OHS) provider throughout the work process. Occupational Health Service providers are independent experts supporting employers regarding the work environment and work-related health issues, such as exposure assessment and occupational health surveillances.

In a Swedish context, the consultants within OHS include e.g., behavioral scientists, ergonomists, occupational nurses, occupational physicians, and work environment engineers. Since the HIW-model is targeting hand-intensive work, the role of an OHS-ergonomist is suitable to support the employers with the occupational health surveillance of workers exposed to hand-intensive work [15, 17]. In Sweden, OHS-ergonomists usually have a bachelor’s degree in physiotherapy (registered physiotherapist), with additional courses in physical ergonomics. Thus, OHS-ergonomists with a background as registered physiotherapist fulfills the requirements stipulated by the Swedish Work Environment Authority and are competent to perform both the exposure assessment and the clinical examination of workers exposed the hand-intensive work (Fig. 1) [15, 17].

Participatory ergonomics implicates that workers are involved in developing and implementing ergonomic measures [20–22]. The HIW-model is intended to support a participatory approach and to involve workers as well as managers in the process [17]. Characteristics of the ergonomists in a participatory process include them initiating and guiding the process, acting as an expert team member, training participants, and being available for consultation [23]. Research about the collaboration between occupational health consultants and representatives from their client companies is scarce. Hence, it is important to explore this area since it can contribute to new knowledge regarding how to improve the collaboration between the parties. Earlier results from the company representatives’ experiences of working according to the HIW-model showed that they valued cooperating with the ergonomist, and they increased their knowledge of ergonomic exposures and its effect on the workers, something which motivated them to act in order to reduce the risk. However, the company representatives also expressed that they were unexperienced in working in a participatory process together with the ergonomist and felt that the HIW-model lacked detailed guidance on the process [24]. In order to develop an optimal process model, which supports the implementation of the occupational health surveillance for hand intensive work, the experiences from the companies’ perspectives, with them having responsibility for the work environment, as well as the perspective from the provider of the occupational health surveillance need to be understood. Thus, the aim of this study was to explore ergonomists’ experiences of the execution of occupational health surveillance for hand-intensive work when following a novel process model. The study focuses on how the ergonomists experience the process model, as well as what factors facilitated or impeded the execution.

## Methods

This study is part of a larger project with the objective of exploring the HIW-model from the perspectives of company representatives (e.g., employers, safety representatives) and OHS-ergonomists. Results exploring the company representatives’ experiences have been published elsewhere [24]. The experiences of the ergonomists were explored with semi-structured individual interviews [25], analyzed with qualitative content analysis [26–28]. The Consolidated criteria for Reporting Qualitative research (COREQ) [29], a 32-item checklist was used to support the reporting of this study. The Regional Ethical Review Board in Uppsala approved the study (project reference number 2017/274).

### Participants

The ergonomists were included based on being ergonomists at the OHS-provider to one of the ten companies that participated in the project. The recruitment of the companies is presented elsewhere [24]. The ergonomists were contacted (by KE or GD) by telephone and e-mail and informed about the study and asked to participate in individual interviews. All ten ergonomists (eight women, two men) accepted and gave both oral and written consent. Their age ranged from 32–57 years, and their working experience as ergonomists within occupational health ranged from 5–34 years. All of them were experienced in clinical examinations as well as in exposure assessments. Nine ergonomists were employed at an external occupational health service, and one was employed at an in-house occupational health service.

### Study context

At each company, a project team, consisting of the ergonomist and the company representatives (e.g., first-line manager; Health, Safety and Environment manager; and safety representative), was formed. The process commenced with a joint start-up meeting, where the company representatives and their respective associated ergonomist attended. The meeting included a presentation of the research project, followed by a presentation of the HIW-model (Fig. 1), which included a thorough description of the components in the model and the overall process, described in Eliasson et al. [17]. The ergonomist was expected to execute the exposure assessment and the medical health checks. A general overview of the exposure assessment was presented at the meeting, including examples of the exposure assessment tools, Hand Activity Level (HAL) [30–32] and Quick Exposure check (QEC) [33], as well as the required content of the clinical examination. The clinical examination was exemplified by the examination protocol, Health Surveillance in Adverse Ergonomics Conditions (HECO) [34]. However, the project teams were informed that these were only examples of tools and that they were free to use other tools for the exposure assessment or follow other examination protocols suitable for the specific assignment in their company. Lastly, at the start-up meeting, each company, together with their respective ergonomist, began planning the execution of the HIW-model. The contracted ergonomists were economically reimbursed according to the existing financial agreements between the company and the OHS provider.

### Data collection

The ten ergonomists were interviewed between eight to twelve months after the start-up meeting. The time and date of the interviews were adapted to the ergonomists’ schedules, and them having completed the exposure assessment and medical health checks, as well as providing a feedback report to the company.

The interview was an individual face-to-face interview [35], held either at the ergonomist’s OHS-company or at the interviewers’ (GD, KE) workplace (Occupational and Environmental Medicine clinic), according to the ergonomists’ wishes. All interviews were conducted by two interviewers, either researchers (TN, CL, PP) or PhD-students (GD, KE). One interviewer was acting as the moderator, actively introducing the questions (GD or KE), while the other interviewer was observing and adding questions when relevant. Both interviewers aimed to create an atmosphere of confidence and to facilitate the participant to speak freely [36].

The interviews were semi-structured and followed an interview guide. The researchers (K.E. and G.D.), both Ph.D. students, registered physiotherapists, and ergonomists with professional experience of OHS, created a draft for the interview guide. The research group discussed the draft several times until reaching a consensus on a final version, which was tested in the first interview but did not result in any modifications thereafter.

The interview focused on the ergonomists’ experiences of working in a process guided by the HIW-model, including their experiences of its different components. Questions regarding the process explored the ergonomists’ participation in the process, e.g., how roles and collaboration with others influenced their work. Examples of questions were: *How did you experience the HIW-model? Tell me about your work with the exposure assessment, *etc*.? Please, tell me about your role in the process? How did you experience the cooperation and collaboration with other parties in the company? Tell me about factors that have facilitated or hampered your work?*

The duration of the interviews ranged from 45 min to two hours. The interviews were audio-recorded and transcribed verbatim in Swedish by a professional transcriber.

### Data analysis

Qualitative content analysis with an inductive approach, according to Graneheim et al. and Lindgren et al. [26–28], was used to analyze the interviews. The interviews were first read through repeatedly to obtain a sense of the whole. The text was divided into meaning units, each composed of several words, sentences, or paragraphs containing aspects related to each other through their content and context. Those condensed meaning units were labelled with codes by the researchers (GD, CL) separately, followed by negotiations on the coding. The coding was made in NVivo 12 software (QSR International Pty. Ltd., Australia). The coding was driven from the manifest data and the material was processed back and forth, which meant that the researchers, repetitively, went back to the interviews to ensure that they were close to the data. The codes were interpreted and compared for differences and similarities (GD, CL), groups of codes were sorted into subcategories, then formulated into preliminary categories. Since data was rich, a higher interpretation was made of the latent content and formulated into themes. The whole analysis was an iterative process, in which GD and CL made the initial analysis into categories and themes. Thereafter, the analysis was presented to AFW who asked questions regarding the sorting and covering of data in categories and themes. The data was further processed and discussion followed until agreement was reached. Then, the analysis was presented and discussed several times with the remaining authors and finally resulted in two main categories and one theme. The analysis process was done in Swedish; for the preparation of the manuscript, the headings of the theme, categories, and quotes were translated into English, which means that quotes have sometimes been slightly amended to facilitate an understanding.

The results were discussed repeatedly in the whole research group. This negotiating process between researchers from different research fields is referred to as triangulation; it is used in this study to ensure credibility of the analysis [37]. All members in the research group have experiences in occupational health and represent different perspectives and backgrounds, such as occupational medicine, physiotherapy, ergonomics, and occupational therapy.

## Results

The findings from the interviews describe the ergonomists’ experiences of the practical work in carrying out the components of the HIW-model: exposure assessment and medical health checks (medical health checks included screening and clinical examination, risk assessment of work influence, and feedback to the employer) (Fig. 1).

The ergonomists’ overall experiences of their work, according to the HIW-model, were summarized in the theme “A joint roadmap supporting a participatory process.” The ergonomists valued the HIW-model since it formed a common picture for everyone involved in the process. The overarching theme comprised two categories: “Clear structure provided by the components” and “The process influenced by collaboration and context” (Table 1). The first category, “Clear structure provided by the components,” describes the ergonomists’ experiences of working in a structured manner, according to the different components in the HIW-model. The second category, “The process influenced by collaboration and context,” describes the experiences of factors regarding collaboration and context, which affected the ergonomists’ possibility to work according to the model. Quotes from the ergonomists are provided in *italics* to illustrate the categories.

**Table 1:** The analytical process resulted in one overarching theme and two categories

Theme	A joint roadmap supporting a participatory process	
Categories	Clear structure provided by the components	The process influenced by collaboration and context

### Clear structure provided by the components

The ergonomists appreciated the systematic manner of working with the model and felt that the components provided structure and clarity regarding what the providers expected them to accomplish. However, the execution was perceived as somewhat challenging, despite a clear structure guiding the work.

*“Working with this model was absolutely useful…I like when the work is systematic… That you start from a problem and connect it both at the individual and the organizational level… It (the model) is well suited for occupational health surveillance of hand intensive work; that is what I think. …”* (Ergonomist 4)

They viewed the component exposure assessment as being the most challenging part of the work process. Various factors affected the exposure assessment, such as workers being absent, or if the work tasks (in the production) were not running at the time of the exposure assessment. Such obstacles could impede the ergonomist’s collection of exposure data and delayed the assessment process. Some of the ergonomists also described insecurity and difficulties regarding selection of appropriate exposure assessment tool(s).

“*The company had chosen which work tasks I should assess, and I was given a certain time and date for the assessment. This meant that I did not get to see the whole work process and also only one worker. This means that you lack information [for the assessment]. So, I felt that the assessment became unreliable; I had to make some assumptions. However, I addressed this shortcoming when I reported [to the company representatives].”* (Ergonomist 6)

The ergonomists used various observational exposure assessment tools. They selected the tools based on what they deemed to be relevant and suitable for assessing the hand intensive work in “their” company. It was also important for the ergonomists that the method chosen could be executed quickly. The tools used were: Quick Exposure Check (QEC) [33], Hand Activity Level (HAL) [30–32], Hand Arm Risk Assessment method (HARM) [38, 39], Assessment of Repetitive Task of the upper limbs (ART) [40], Rapid Upper Limb Assessment (RULA) [41], Key Indicator Method I (KIM I) [42], Key Indicator Method III (KIM III) [43], and Comprehensive Workplace Risk Assessments [44]. The ergonomists pointed out that the exposure assessment was facilitated if they could video record the work tasks. However, video recordings were not allowed in all companies, due to the respective company’s security restrictions.

Some ergonomists expressed that it was important to supplement the exposure assessment with information about stress and recovery, in order to get as complete assessments as possible of hazardous exposures related to WRULDs.

*“I think the factors stress and recovery are very important [to consider], and they are not included in the risk assessment [exposure assessment tool]. These factors can affect symptoms in the body. Sometimes, you do not ask about stress because you do not know how to handle the answers [in a risk assessment]. The response to stress is so individual. I think we need to learn more about stress and recovery*.” (Ergonomist 3)

For the component screening and clinical examination, the examination protocol Health Surveillance in Adverse Ergonomics Conditions (HECO) [34] was used by nine ergonomists. One ergonomist performed a “standard” clinical examination. The ergonomists thought that the HECO was suitable and convenient. They expressed that the protocol was easy to use and that it provided support in the examination. The report summarized that using HECO was valuable, as it gave a clear picture of the prevalence of musculoskeletal disorders in the examined work group. Furthermore, the HECO makes it possible to compare the results from the examined work group (illustrated in bar charts) to the results from other workers in other work sectors. The report served as a professional presentation when reporting the results to the company’s representatives.

*“The HECO was very comprehensive and covered many parts. The individual meeting is incredibly important since you get descriptions from different people [about the work]. The HECO-examination was done in a room that we [OHS-consultants] usually use at this workplace. The examination took about 45 minutes per person. I think that those who came to the examination experienced it as very positive.”* (Ergonomist 10)

The ergonomists stated that it was important to include all exposed workers from a work group in the screening and the clinical examination to get an overall picture of the effect of the assessed exposures. In some companies, the contact person only selected workers with already known, and sometimes severe, musculoskeletal disorders from the exposed work group, for the screening and clinical examination; hence, the results could not be generalized to the whole work group.

*“It was only a few workers participating in the clinical examinations and those who participated had been working for a very long time at the company and with poorer ergonomic conditions from previously. They probably had a lot of disorders already, which I conclude were caused by the work. But now, I tried to assess whether the present work tasks that I had assessed could have caused those problems… I would absolutely have preferred to examine all the [exposed] employees.” (Ergonomist 4)*

The ergonomists expressed that their goal with the feedback to the employer was to increase the risk awareness by explaining how the exposures affect the workers’ health, emphasizing the need for risk reducing actions. The ergonomists described that providing the feedback, both in a written report as well as an oral presentation during a subsequent meeting, made it easier to get the message through to the company representatives and to initiate a dialogue. This approach was used by most of the ergonomists. The feedback included the results of the exposure assessment and the HECO report on a group level. However, if the work group was small, it was difficult to ensure the workers’ anonymity, which complicated the feedback phase. The ergonomists perceived that the company appreciated when proposals for risk reducing actions were included in the feedback. Some ergonomists described that the feedback directly led to discussions with managers and workers, where risk reducing actions, planning of education, or re-organization were addressed. However, some ergonomists thought it was difficult to suggest risk reducing actions that could guarantee an exposure reducing effect.

*“It is important that you also give feedback orally because you formulate it in a different way and you can provide more details that are not included in the report, which can be important aspects. And they can ask questions, ‘what does that mean’ and you can answer it. It is, of course, important that the manager is involved in the feedback”.* (Ergonomist 10)

Furthermore, feedback was also given directly to workers during the ergonomist’s work with exposure assessment and the clinical examination. The individual feedback contained tips regarding work technique and rehabilitation/treatment of disorders.

### The process influenced by collaboration and context

Interviews revealed that collaboration between the different parties, as well as the different contextual settings, affected the ergonomists’ work process, both when it came to facilitating and as a hindrance. Affecting factors regarding collaboration were related to lack of clarity in expectations and communication between different functions (e.g., the ergonomist and the contact person) in the process. Affecting factors in the context were related to the companies’ routines concerning work environmental issues, uncertainties regarding billing and costs, and the ergonomist’s intra-organizational support from his/her employer (the OHS provider), such as access to IT-resources.

The ergonomists experienced that the work process was facilitated by the start-up meeting, in which the ergonomist, together with the company representatives, reviewed the HIW-process and jointly started the planning by setting a schedule for the execution. One ergonomist described that they also planned for several feedback meetings in their project team (consisting of Health, Safety and Environment manager; safety representative; and first-line manager) during the process, namely one feedback meeting after each component. The ergonomist felt that these regular meetings facilitated the process, since information was dispersed within the team, and the meetings kept the work process going.

*“We tried to have meetings frequently during the process. It was very good; it was the contact person who took responsibility for this…That was a strength and very good, something to pass on to others regarding this work process…”* (Ergonomist 1)

Involving the safety representatives in the process was also described as valuable, since they possess knowledge regarding the exposures but also knowledge regarding organizational factors.

*“The safety representative had quite a lot of information, which might have been good to include in the assessment. So, I believe it would have been great to be instructed to involve the safety representative more in the process.”* (Ergonomist 2)

All ergonomists highlighted the contact person at the company as very important for the overall process. The ergonomists described that the contact person facilitated and supported collaboration between all those involved. A collaboration, *“side by side”* (Ergonomist 4) with the contact person and the group of workers, was the basis for a frictionless process. In companies where the collaboration between the ergonomist and the contact person was lacking, the process was hampered. An example of this was that it took time for the ergonomist to get in contact with and access the workplace for conducting the exposure assessment or to get in contact with the workers for the clinical examinations.

The ergonomists explained that a contact person should have the mandate to manage the process, be interested in the process, and be in close connection with the workplace and the workers (e.g., not be situated in an office in another town far from the workplace). Furthermore, the contact person should have skills to communicate and support the process for all involved parties (ergonomist, workers, safety representatives, and managers) and have an understanding of their different perspectives. Moreover, practical support, such as helping the ergonomist with access cards and booking a room at the workplace, was needed.

*“The most important success factor was to have a “good” contact person within the company, one who understands what is going on and how we want to work all the way…I had a lot of help from the contact person, who booked the premises and time…I got there and did my job as I was supposed to…”* (Ergonomist 4)

There were examples of the process being hampered due to the manager, safety representatives, or workers being too busy, passive, or reserved. Some ergonomists felt that the lack of information given to the workers prior to the clinical examination hampered the execution at times, and they highlighted the importance of ensuring that sufficient information had been given to the workers in advance. One ergonomist described a group of workers that was reserved and raised questions like *“…who ordered this…”, “…what do we get out of this…”* (Ergonomist 4). Another ergonomist described that the workers misunderstood the aim of the occupational health surveillances due to language issues, and therefore were unwilling to participate in the medical health check. The ergonomists managed these situations by adapting the process, for example, by organizing a separate meeting with these workers, in order to create a relationship with them and thoroughly explain the aim of the occupational health surveillance. This contributed to the workers being more interested in the process and made it possible to continue the collaboration with the workers.

*“I think it was great that I, as an ergonomist, informed the workers. Because it might not be that easy for an employer to really explain what is going to happen, how, and why. And just to show who you are is probably really great…They had the time to pose questions to me as well. So, I think it was a good to add the meeting”.* (Ergonomist 9)

*“When the workers came to the clinical examination, many of them did not know why they were there. Perhaps the information from the company was not enough…It would have been helpful with written information (to the worker) including screening questions prior to the examination...”* (Ergonomist 5)

However, not all ergonomists felt secure in their role and function in the process, which could be related to the ergonomists’ as well as the company representatives’ lack of experience with collaborating in similar processes. Some ergonomists experienced uncertainty regarding costs and how to debit for unpredicted events that could occur during the process (e.g., due to sickness, organizational changes in the company). This brought insecurity as to how much time the ergonomist could spend on the work. Several ergonomists emphasized the importance of defining a financial agreement before the start of the work process.

Other contextual factors affecting the work process were intra-organizational factors from their own employer (the OHS provider). The intra-organizational factors affecting the ergonomists’ work in the HIW-process were: support from the own management, access to technical devices (mobile camera, computer), software and IT-support as well as the possibility to ask other ergonomist colleagues for advice, for example, regarding methodological issues, such as tools for the exposure assessment.

## Discussion

The main result: *A joint roadmap supporting a participatory process* shows that the ergonomist valued the HIW-model since it guided a structured work process and provided support and preconditions for participatory collaboration in the process. Collaboration with the contact person at the company was an important factor in it being successful. Factors impeding the ergonomists’ work were difficulties with exposure assessments, collaboration difficulties, lack of information regarding the health of an entire work group (or interpreted results for a small group of workers), insecurity regarding debiting and intra-organizational structures interfering with the work.

The main results of this study concerning the work process according the HIW-model are in line with what the company representatives (e.g., managers, safety representatives) perceived, in their role as being responsible for the work environment. For example, both the ergonomists and the company representatives appreciated working according to the HIW-model [24]. Their experiences are mainly consistent with each other, even though the ergonomists and the company representatives have dissimilar roles and functions in the process. Both parties appreciated being able to follow a structured joint process [24]. One goal for the ergonomists was to contribute to an increased risk awareness in the companies. According to the company’s representatives, this was achieved, which highlights that the process contributed to clarifying the effect that adverse exposures have on workers’ health [24]. Both studies reveal the importance of a joint start-up meeting at the beginning of the occupational health surveillance process [24]. The aim of this meeting should be to thoroughly plan the process, e.g., for regular checkpoints, practical issues, and the appointment of a contact person (or process leader) within the company who should be able to support the ergonomist. Halonen et al. [45] evaluated the facilitating factors for the collaboration between the occupational health service providers and the employers and described that effective collaboration consisted of: shared goals, reciprocity, frequent contact, and trust. Similar factors are mentioned by the ergonomists in the present study. Earlier research has reported positive health effects when workers, managers, and an ergonomics facilitator work together [46]. It is described that a team with workers, supervisors, and specialists represents key actors in a participatory ergonomic process [47], as was intended in the HIW-model.

This study focuses on the specialists, namely the ergonomists. Their experiences divulge information about *how* to act in the specialist role within the process, but also which areas the ergonomists need to strengthen in order to be successful in the process. The ergonomists emphasized the importance of a smooth collaboration with a dedicated contact person for a successful process. This is similar to what Burgess-Limerick et al. [48] reported, highlighting the importance of a person onsite, as they describe as a site champion, who drives the process. That person should have easy access to, and support from, the management to proceed with projects [48]. Additionally, results from the present study further add that the contact person onsite should also have close access to and communication with the workers.

Previous research reports that OHS ergonomists often enter the risk assessment process after the identification of musculoskeletal disorders in workers and that a systematic approach in risk assessment assignments, in general, is lacking [49, 50]. This study explored the ergonomists in a partly new role, in which they entered the risk assessment process early on and where they interconnected the exposure assessment with medical health checks of exposed workers, in order to make a total assessment as to whether the work influenced the risk for WRULD. Different exposure assessment tools were used among the ergonomists; moreover, it is important to use reliable tools for risk assessments as they result in trustworthy assessments, as well as facilitate dialogues with stakeholders at client companies [51, 52]. However, the ergonomists indicated the need to develop their knowledge and skills regarding exposure assessments, for example, which tools are appropriate for the assessment of hand-intensive work. Furthermore, the ergonomists raised the importance of including workers’ descriptions of stress and recovery as factors to consider in their risk assessment. This is in line with Macdonald et al. [53] and Oakman et al. [54], arguing for the necessity to have a holistic approach and addressing risks from all relevant hazards (physical as well as psychosocial) in order to help with the prevention of musculoskeletal disorders. Most ergonomic exposure assessment tools focus on the physical aspects of work and concentrate on specific body regions [55]. In the present study, the ergonomist assessed the physical exposure but also met the workers individually in the clinical examination, which means they had an opportunity to ask the workers about affecting psychosocial factors. However, this was not done in general, which indicates that the ergonomists need to increase their knowledge on how to combine exposure assessment of the biomechanical/physical factors with the assessment of psychosocial factors.

Results from the study regarding the company’s representatives show that not all of them were used to collaborating with an ergonomist in a risk assessment process [24]. Other studies report that company managers associate ergonomics mainly with health issues, and there is a lack of knowledge about the ergonomists’ broad competence [50, 56, 57]. This highlights the importance of strengthening the ergonomists in their role as guides throughout the process, assisting the company in incorporating the HIW process into the overall risk management process. A goal with the cyclic “plan-do-act-check” structure in the HIW-model is that the process should be recognizable for integration in the risk management system [17]. However, the ergonomists do not explicitly reflect on this in the interviews, which might indicate an uncertainty regarding the ergonomist’s role and the need for ergonomists to increase their confidence regarding their own skills and value as an expert for the company. Research have shown that if organizations follow a Plan-Do-Act-Check process model, then they have increased possibilities for maximizing invested money for a safer working environment [58].

When working according to the HIW-model, it is important that the ergonomist has a macro-ergonomics system perspective so that the occupational health surveillance does not become solely an isolated event targeting the individual. The aim is that the HIW-model should support a sustainable reoccurring process, well incorporated into the regular OHSM system. Hence, OHS ergonomists need to increase their competence to guide a participatory process. Such skills include, for example, problem-solving, collaborative approaches, and an ability to build alliances with stakeholders. Several studies have reported that such skills are important in order to put ergonomics issues on the companies’ agenda and to make changes that will improve the workplace ergonomics and prevent musculoskeletal disorders [50, 59, 60].

A variety of contextual factors can affect a work process, which was also apparent in this study. The ergonomist’s work was affected by his/hers own OHS-organization as well as the company’s organization, where the HIW-model was tested. The intra-organizational factors (within the OHS organization) are important for the implementation and stimulation of new working methods, such as the HIW-model. Studies have shown that even though OHS-employees are motivated to implement new working methods, the management does not stimulate the employees in their pursuit [61, 62]. Even though this study mostly focuses on the ergonomist’s role and function in the tested work process, the ergonomists raised concerns regarding the lack of intra-organizational support. It is important to consider how the OHS-organization can support the employees in their work; however, this is outside the scope of the present study.

### Strengths and limitations

We were interested in the ergonomists’ experiences of the HIW-model and used interviews, as they are considered to be an appropriate method in order to explore people’s experiences [35]. Several methods could have been used for analysis of the interviews. A qualitative content analysis according to Graneheim et al. and Lindgren et al. [26–28] was choosen, hence it was important to explore the experiences from the users of the HIW-method in different context. Since the context is emphasized in qualitative content analysis we found the method suitable. An inductive, data-driven approach was used in analysis since we wanted to ensure that all information from the users were included in analysis, if a deductive approach had been used, e.g., if the coding was based on the HIW-model, could have meant missing information. The results are based on ten ergonomists’ experiences, associated with the ten participating companies in the study. Hence, *all* ergonomists who had experience of the HIW-model were interviewed. The quite small number of participants could potentially be a limitation in the study, however as the study focus on experiences of a very specific method we conclude that the number of participants is sufficient. Even though the context varied in the different companies, the experiences among the ten ergonomists were similar, regarding the HIW-model, the ergonomists’ role in the process, and facilitating and hindering factors.

The interviewers (GD and KE) had previous experiences of working as an ergonomist in the OHS, which might have contributed to the informants, consciously or unconsciously, withholding criticism, or assuming that there was a mutual understanding regarding certain issues, and therefore did not express themselves as clearly as they might have done to an uninitiated interviewer [26, 27]. However, it might also have facilitated the interviews, as the common background contributed to a pre-understanding of the ergonomists’ practice. Hence, to ensure the study’s trustworthiness and quality, several researchers (all contributing authors) with different backgrounds regarding experience of OHS, as well as qualitative analyses, were involved in the interviews and in the data analysis [63].

This study explores ergonomists’ experiences as providers of occupational health surveillance, targeting hand-intensive work according to the HIW-model. The legislation, an occupational health surveillance targeting hand intensive work, is, to our knowledge, quite unique. It is, therefore, unclear if the findings can be generalized to OHS-ergonomics practitioners outside of Sweden. However, worldwide, there are different legislations regarding occupational health surveillance, and the process might be transferable to other surveillances targeting other exposures, such as vibrations. Furthermore, in the broader perspective (beyond legislative regulations and exposure levels), the findings in this study contribute to knowledge regarding factors influencing collaboration in a joint work process between the work environment consultants and the company’s representatives. Factors, such as having a designated contact person at the company, a clear schedule for the work process, and anchoring of the process with the employees should be important to consider in other participatory work environment interventions.

## Conclusions

This paper provides information regarding important factors that facilitate and hinder the implementation of the HIW-work process from the ergonomists’ (the providers’) perspective and adds knowledge to the previously explored perspectives of company representatives [24]. The model for a systematic process facilitated ergonomists’ work and cooperation with their client company, and a visualization of the work process contributed to a joint image of the common work. Prior to implementation, the HIW-model needs to be developed and accompanied by guidelines with information relating to the process. This should include, for example, a description of the purpose and outcome of the start-up meeting, feedback after exposure assessments, and a description of the roles/functions of the different parties involved.

## Supplementary Information

**Additional file 1.** Interview schedule.

## Data Availability

A unique code has been assigned to each interviewed ergonomist. Thereby, the data from the transcripts are anonymous and can only be tracked back by an identification log. This identification log can only be accessed by the primary researcher (KE). Transcripts of interviews (anonymous format) and the signed informed consent forms have been stored on a secure network drive for 10 years. Digital audio files have been stored on a secure network drive in an anonymous format for 10 years. The anonymized data generated and/or analyzed during the current study are available from the corresponding author upon reasonable request.

## Abbreviations

WRULD: Work-related upper limb disorders
HIW: Hand-intensive work
OHSM: Occupational health and safety management
OHS: Occupational health service
HECO: Health surveillance in adverse ergonomics conditions
COREQ: Consolidated criteria for reporting qualitative research
HAL: Hand activity level
QEC: Quick exposure check
HARM: Hand arm risk assessment method
ART: Assessment of repetitive task of the upper limbs
RULA: Rapid upper limb assessment
KIM: Key indicator method
