# Supplementary material for: Ergonomists’ experiences of executing occupational health surveillance for workers exposed to hand-intensive work: a qualitative exploration
Source: BMC Health Serv Res. 2022 Oct 1;22:1223. doi: 10.1186/s12913-022-08601-2 (PMC9526985; doi:10.1186/s12913-022-08601-2)
Supplement: Supplementary file 1 — Additional file 1. Interview schedule. [file 12913_2022_8601_MOESM1_ESM.docx]

Interview schedule

| Dates for the start-up meetings: | Ergonomists at the start-up meetings, representing companies no.: | Dates for interviews with the ergonomists |
| --- | --- | --- |
| 2017-10-03 | 4,5,6,7 | No. 4 – 2018-09-17  No. 5 – 2018-10-09  No. 6 – 2018-10-09  No. 7 – 2018-10-01 |
| 2017-10-16 | 1,2,3 | No. 1 – 2018-09-06  No. 2 – 2018-09-05  No. 3 – 2018-09-05 |
| 2018-01-22 | 8,9,10 | No. 8 – 2018-09-24  No. 9 – 2018-10-02  No. 10 – 2018-09-25 |
